# Spinal Paralysis

**Published:** 1889-02

**Authors:** A. McNeil

**Affiliations:** San Francisco, Cal.


### SPINAL PARALYSIS.

A. McNEIL, M. D., SAN FRANCISCO, CAL.

Jan. 25th, 1888, I was called to see Fred. Greenland, of No. 1 Jackson St. He is about thirty-five years of age, German, tall, muscular, and fair, but now reduced. In February, 1885, was thrown from a horse, and the upper part of femur split. In three months after the accident returned to his work, but was compelled to give it up in a week. In fourteen months after his fall his back became sore and painful, which continued till a well-marked anterio-posterior curvature forming an angle about midway of the scapulæ formed. This no longer is painful. He is compelled to walk on crutches, on which he bears heavily and moving one leg forward at a time. He suffers so much as to almost deprive him of sleep from pain in lumbar region around to the right groin and in both legs; sensation unimpaired. He is a hard drinker. His pains are aggravated by rest, although motion is painful.

I gave him Rhus-tox.$^{30}$ in water for twenty-four hours, a teaspoonful every two hours; next day Sac. lac.

February 2d.—Rhus-tox.$^{cc}$ a powder dry. He has improved, but is again suffering more from the pain.

February 10th.—Rhus-tox.$^{cc}$ one powder dry.

On the 21st, Rhus-tox.$^{500}$ dry, one powder. He has improved, but the improvement has stopped.

March 6th.—Rhus-tox.$^{m}$ in the same way.

Did not see him till May 13th. He has improved remarkably. Gave him Rhus$^{35m}$ one powder.

He turned up on September 18th. Walking with a cane. Still has some stiffness on sitting or lying quiet for a time.

Rhus$^{75m}$ one powder, but otherwise walks nearly in a normal manner. He carries a cane, but shouldered it and marched like a soldier. During all of this time he has kept up his drinking in spite of my remonstrances.

Is he cured or was it a case of recovery? A reputable allopathic physician said before I began with him that he never could stand unaided on his feet. And then the long continuance of the disease and the immediate improvement on my assuming his case and the beneficial effect which followed in every case after I renewed the medicine proved it. His drinking, too, was almost certain to stand in the way of the efforts of the *vis medicatrix naturæ*.

That Rhus-tox. was indicated was evident, although, from its ætiology, Arnica might be thought of; yet the modalities pointed to Rhus and not to Arnica. But it may be asked why did I give the potencies I did. I began with the 30th, and did not repeat until the action was exhausted, and then gave a higher potency. Not very long after I began to practice scientific therapeutics, I found that patients ceased to respond to the potency of the remedy which I had given with good effect before, although the symptoms still indicated that remedy. But, on going higher, the remedy would again do good work. I afterward learned that Lippe and other Hahnemannians followed that rule in the administration of remedies. I now save much time by never repeating a potency, but always going higher.

